# Distinct phenotypic behaviours within a clonal population of *Pseudomonas syringae* pv. *actinidiae*

**DOI:** 10.1371/journal.pone.0269343

**Published:** 2022-06-09

**Authors:** Nuno Mariz-Ponte, Emil Gimranov, Rute Rego, Luísa Moura, Conceição Santos, Fernando Tavares

**Affiliations:** 1 Biology Department, Faculty of Sciences, University of Porto (FCUP), Porto, Portugal; 2 LAQV-REQUIMTE, Biology Department, Faculty of Sciences, University of Porto (FCUP), Porto, Portugal; 3 CIBIO-Research Centre in Biodiversity and Genetic Resources, In-BIO-Associate Laboratory, Campus de Vairão, University of Porto, Vairão, Portugal; 4 CISAS—Centre for Research and Development in Agrifood Systems and Sustainability, Instituto Politécnico de Viana do Castelo, Viana do Castelo, Portugal; 5 BIOPOLIS Program in Genomics, Biodiversity and Land Planning, CIBIO, Campus de Vairão, Vairão, Portugal; University of Trento, ITALY

## Abstract

Bacterial canker of the kiwifruit caused by the etiological agent *Pseudomonas syringae* pv. *actinidiae* is the most severe disease in kiwifruit production. Since 2008 a hypervirulent Psa biovar 3 has spread rapidly worldwide. Different genomic and phenotypic approaches have been used to understand the origin of the dissemination and geographical evolution of populations associated with this pandemic. This study aimed to characterize the genetic and phenotypic diversity of 22 Psa isolates collected in different regions of Portugal between 2013 and 2017. Genotypic and phenotypic characterization was based on Multi-Locus Sequence Analysis (MLSA), motility, IAA production, Biolog GEN III, and copper sensitivity. No polymorphisms were detected for the concatenated sequence (1950 bp) of the housekeeping genes *gltA*, *gapA*, *gyrB*, and *rpoD*. Results support the analysed Portuguese Psa isolates (2013–2017) belonging to Psa3, and MLSA indicates high genetic clonality and stability of these populations. The phenotypic analysis through Biolog revealed a heterogeneous pattern in the Psa collection and its position in the Pseudomonas complex. This heterogeneity reflects a genomic diversity that may reflect distinct adaptive trends associated with the environmental conditions and widespread. The Portuguese Psa collection showed no resistance to copper. This information is relevant to kiwi producers that predominantly use Cu-treatments to control kiwifruit bacterial canker.

## Introduction

*Pseudomonas syringae sensu lato* includes important plant pathogens responsible for high losses in a wide taxonomic range of plant crops. Phytopathogenic *Pseudomonas syringae* includes more than 60 pathovars, which have been clarified based on phylogenomic analysis [1]. In the last decades, the kiwifruit bacterial canker, caused by *Pseudomonas syringae* pv. *actinidiae* (Psa), has been affecting the world production of kiwifruit [2, 3]. This pathogen, which was first isolated in Japan [4], is known to infect *Actinidia deliciosa* (green-fleshed kiwifruit) and *A*. *chinensis* (yellow-fleshed kiwifruit), acknowledged as the two most economically relevant species of kiwifruit worldwide [5, 6]. Psa usually grows on epiphytic surfaces of kiwifruit flowers and leaves. After this phase, it enters the plant via natural openings, such as stomata, flowers, or wounds [7, 8].

The symptoms of Psa infection are characterized by bacterial ooze in the trunks, browning or darkening of vascular tissues, development of wilting and blight symptoms in leaves, necrotic spots on leaves contoured by yellowish halos, dark coloration, and detachment of buds and flowers [5, 9–11].

Psa is included in the A2 list of quarantine pathogens by the European and Mediterranean Plant Protection Organization (EPPO) [12]. After its first report in Japan in 1984, this disease was later identified in Korea, and the most virulent biovar (Psa3) appeared in 2008 in Italy [4, 13, 14], raising concerns about its impact in Europe. The Psa3 identified in Italy and in many other countries is highly virulent, leading to successive outbreaks worldwide [2, 15–17]. In Portugal, the occurrence of Psa was first reported in 2010 in kiwifruit orchards of the northern regions of Entre-Douro and Minho [15].

Psa is divided into biovars, namely the Psa1, Psa2, Psa3, Psa5, and Psa6, according to their biochemical behaviour, genetic differences, and distinct virulence [10]. Biovar Psa3 is the most virulent and is associated with the recurring outbreaks of kiwifruit bacterial canker observed in New Zealand and Europe, including Portugal [18, 19]. This biovar originated from China, and independent transmission events were responsible for its pandemic spread to other regions [17].

MultiLocus Sequence Analysis (MLSA) is a sequence-based method of genotyping for inter- and intra-species discrimination in bacterial taxonomy and is commonly used to characterize different isolates in the Pseudomonas genus spp. [1, 20, 21]. The analysis is based on single-nucleotide polymorphism in sequences of four to ten housekeeping genes, being the genes *DNA gyrase subunit B* (*gyrB*), *Glyceraldehyde-3-phosphate dehydrogenase A* (*gapA*), *RNA polymerase sigma factor D* (*rpoD*), and *Citrate synthase* (*gltA*) the most used for Psa characterization [22–25]. Different databases were developed for MLSA on *Pseudomonas* spp. such as PseudoMLSA [21]. MLSA has also been the most used approach in genotypic Psa characterization with the capability to distinguish different Psa biovars [3, 26–28].

Despite the genotyping tools are increasingly gaining importance in bacterial taxonomy, it is well acknowledged that a multiphasic approach, including a phenotypic characterization, is indispensable in bacteria identification. The phenotyping provides information on the strains’ metabolism and resistance to antibiotics, which is essential to disclose bacterial adaptation traits. For *Pseudomonas syringae* complex the current phenotypic-based method such as Biolog Microbial Identification Systems (GENIII plates) has been used for species identification and characterization [29]. This phenotyping platform, together with other traits namely IAA production, mobility, and copper resistance, can contribute to characterizing the virulence of Psa. Indol-3-acetic acid (IAA) has been described as a virulence factor in pathogenic Pseudomonad species [26, 30–32]. IAA plays a role in pathosystems after infection by Pseudomonas spp. This role involves controlling the host response by internal phytohormone regulation and thus regulates the plants’ sensitivity during the infection process [31, 32]. IAA acts as a microbial signal molecule in *P*. *syringae* pv. *tomato* suppressing the host defenses and upregulating gene expression of virulence genes [33]. In *P*. *savastanoi*, the high expression of type-III and type-IV effector genes was related to IAA, which has a critical signaling role in this pathogen infection [34]. Chilean Psa3 isolates showed some variability in their motility and in the levels of IAA production. These results led the authors to hypothesize that IAA has an important role in Psa virulence [26]. These results support the interest in quantifying these phenotypic traits to characterize possible more virulent strains in Psa biovar 3. Bacterial motility, associated with high secretion of biofilm components, has been reported in some phytopathogens, such as *Pseudomonas* spp., improving their colonization and virulence traits [35–37]. In Psa, the mobility and biofilm traits have been related to the successful colonization of the host plants [38].

Copper-based formulations are the most used field treatments against Psa, in both prophylactic and therapeutic applications [39]. However, the efficiency of these treatments has been decreased, and mechanisms of resistance to copper were identified in some Psa strains. These findings raise concerns about the recurrent use of Cu-based treatments [40–42] and call for the need to monitor resistance to copper resistance in Psa isolates. Psa isolates showing Cu-resistance were found in Japan, with Minimal Inhibitory Concentration (MIC) values between 1.8 to 2.4 mM of CuSO4 [43]. Also, New Zealand strains recorded MIC values of 1.2 mM [41], both using glycerol minimal medium (MYG). On the other hand, Chilean Cu-susceptible Psa strains revealed a MIC value of 0.075 mM [26] using tris minimal medium (TMM).

Conventional genetic approaches to the Psa3 population in Europe supported in the last decade a clonal community as proposed by Firrao et al. [44]. Also, after the first report of Psa3 in Portugal in 2010 by Balestra et al, [15], other authors supported a highly conserved population structure of Psa3 [18, 45]. Ciarroni et al, [46] identified a different pattern through fragment analysis among two Psa strains from Portuguese orchards. Genetic diversity in Psa was also found by Box-PCR associated with seasonal and spatial dynamics from four Portuguese orchards [19]. This study aims to report the genetic and phenotypic diversity of Psa isolates from Portuguese orchards of eight distinct municipalities (North of Portugal) isolated between 2013 to 2017. The genetic and phenotypic diversity will be assessed by MLSA, motility, IAA production, Biolog, and copper sensitivity.

## Materials and methods

### Bacterial strains, identification and culture conditions

Psa strains were isolated by L. Moura between 2013 and 2017 from kiwifruit orchards of *A*. *deliciosa* geographically located in different regions in the North of Portugal. Isolates were identified as Psa by duplex-PCR as detailed in the S1 Table. Bacteria were grown on solid (1.5% agar) King’s B medium (KB) at 28°C for two days for colony morphology analysis [47], and pure colonies were grown in KB broth at 28°C at 180 rpm for 16 hours. DNA was extracted from cells pellet using E.Z.N.A.® Bacterial DNA purification Kit (Omega Biotek, USA) following the manufacturer’s instructions. For molecular identification of the 22 isolates, a duplex-Polymerase Chain Reaction (PCR) was performed as recommended by EPPO guideline [47] and recently demonstrated by Loreti et al. [48] as a powerful method for Psa identification. The primers used for identification are in the S2 Table: the primers KN-F/KN-R [49] and AvrDdpx-F [50] are specific for *omp1* (*outer membrane protein 1*) and *avrD1* (Effector) genes, respectively. The duplex-PCR reaction was performed with Dream Taq (Thermo Fisher Scientific, USA) using 10 ng as a DNA template, with 3 minutes for initial denaturation at 95°C, 35 cycles of 30 seconds of denaturation (95°C), 30 seconds of annealing (58°C), and 30 seconds of extension (72°C) followed by 7 minutes of final extension. Water was used as a negative control, and the Psa3 reference strain CFBP 7286 (isolated in Italy in 2008) was used as a positive control. PCR products were separated by gel electrophoresis (1% agarose gel, w/v) in Tris-Acetate-EDTA (TAE) 1% buffer, and products were stained with Xpert Green DNA Stain (GRiSP, Portugal). Gel images were obtained by Gel-Doc (Bio-Rad, USA). To confirm those bands, *omp1* and *avrD* amplicons of CFBP 7286 and P84 (used as an example of Psa isolates from Portugal) were isolated from the gel and purified with Illustra GFX™ PCR Gel Band Purification Kit (GE Healthcare, USA) and sequenced (STAB Vida, Portugal) by the Sanger sequencing method.

#### Molecular characterization by Multi-Locus Sequence Analysis (MLSA) of Psa isolates

After confirming the Psa isolates by duplex-PCR (S1 Fig), four housekeeping genes were chosen for MLSA analysis according to previously described protocols [26–28]. The genes *gapA*, *gltA*, *gyrB*, and *rpoD* were selected, coding respectively for the products *Glyceraldehyde-3-phosphate dehydrogenase A*, *Citrate synthase*, *DNA gyrase subunit B*, and *RNA polymerase sigma (70) factor*. Primers were chosen for MLSA according to Sarkar and Guttman, [22] (S3 Table). Bacterial DNA previously extracted by E.Z.N.A.® Bacterial DNA purification Kit (Omega Bio-tek, USA) was used for PCR amplification with selected primers, DreamTaq (Thermo Fisher Scientific, USA), and dNTPs (GRiSP, Portugal) under the following conditions: 3 minutes of initial denaturation at 95°C and 35 cycles of 30 seconds of denaturation at 95°C, 30 seconds annealing at 60°C (except for *rpoD* amplification– 65°C), and 45 seconds of extension at 72°C, and 7 minutes of final extension. Amplification products were visualized in agarose gel (1%, w/v) stained with Xpert Green DNA Stain (GRiSP, Portugal) after electrophoresis (90v by 30 minutes) in Gel-Doc (Bio-Rad, USA). Positive (CFBP7286) and negative (sterile and deionized water) references were used for all reactions and electrophoresis with a suitable molecular weight ladder (GeneRuler 1 kb Plus DNA Ladder). Bands were extracted from the gel and purified with GFX™ PCR DNA and Gel Band Purification Kit (GE Healthcare, USA). Amplified genes were submitted to Sanger sequencing at STAB Vida (Portugal). Sequences were confirmed by forward and reverse sequencing, and partial genes were uploaded on NCBI (S4 Table). These sequences were concatenated with the following order *gapA* [588 bp], *gltA* [433 bp], *gyrB* [480 bp] and *rpoD* [449 bp]. Then, concatenated sequences (1,950 bp) were used to construct a Bayesian phylogenetic tree (Geneious, USA). Other Pseudomonads strains were joined for the analysis (S5 Table). Gene sequences of these strains were obtained in the National Center for Biotechnology Information (NCBI) and aligned with CFBP 7286 (reference strain Psa biovar 3 –Italy 2008), adjusted to its size, and concatenated for MLSA analysis.

### Phenotypic characterization by Biolog GEN III microplate

The Biolog (Biolog, USA) system with GEN III microplate was used for the phenotypic characterization of the Psa isolates (S1 Table) and other strains of the Pseudomonad species complex (S6 Table). All strains were grown in KB medium (agar 1.2%) for 48 hours. Fresh colonies, isolated from pure cultures, were chosen to prepare the Biolog inoculum in the Inoculating Fluid following the manufacturer protocol [51, 52]. The turbidity was adjusted to 90% as recommended for default protocol and 100μL of the inoculum was distributed in each well in the Biolog 96-well microplate. The plates were kept at 28°C for 7 days, and daily records were made in a Biolog MicroStation Reader (Biolog, USA). The experiment was independently replicated (two times) following the same method. Among the replicates for each well tested, results classified as borderline and negative means negative, and borderline with positive means positive. Results were recorded and analysed with the R software using the FactoMineR package for PCA and hierarchical classification analysis.

### Indole acetic acid (IAA) production in Psa isolates

The production of indols such as IAA was quantified according to Gordon and Weber [53] using Salkowski’s reagent (solution of 12g of FeCl3 per liter in 7.9 M H_2_SO_4_). This solution reacts with IAA producing a pink color, due to IAA complex formation by reducting Fe3+ [54]. According to Flores et al. [26], Psa was grown in Luria-Bertani (LB), overnight at 25°C and 180 rpm. The OD_600_ was adjusted to 0.1. Then, 20 μL of the diluted inocula were used to inoculate a new tube with 4 mL of LB medium supplemented with Trp (2 g L^-1^). The isolates were grown for 24 hours in an incubator at 180 rpm at 25°C. After the incubation, OD_600_ was measured for all isolates, and bacterial cultures were centrifuged at 10,000 rpm for 10 minutes. Cell-free supernatants were collected and 0.5 mL of Salkowski reagent was mixed with each sample. The mixture was kept in the dark for 30 minutes and, after incubation, the absorbance of the samples was measured at OD530 in Multiskan GO (Thermo Fisher, USA). The IAA concentration for each sample was determined using a standard curve of indoleacetic acid (0–30 μg mL^-1^). The data were normalized with the cell density measured previously. The experiment was replicated three times in different periods. Statistical analysis was determined by one-way ANOVA in GraphPad Prism 7.

### Bacterial motility assay

Motility was evaluated following Flores et al. [26] procedures for Psa, using a semi-solid LB medium with 0.3% agar. The bacteria were grown for 20 hours and the OD600 was measured and adjusted to 1.3. After adjusting the cell density, 2 μL were inoculated in the centre of the plate for swimming mobility assay. The plates were kept in the incubator at 30°C for 72 hours. The motility into the plates was recorded in a Gel-Doc (Bio-Rad, USA), and the area (mm^2^) was measured using the ImageJ software. Three biological replicates were performed, and the statistical analysis was performed using one-way ANOVA in GraphPad Prism 7 (USA).

### Copper sensitivity

The determination of copper sensitivity was performed using the Tris Minimal Medium (TMM) supplied with a different range (0 to 125 μg) of copper sulfate (CuSO₄) according to Flores et al. [26]. Psa isolates from pure colonies, grown for 48h, were inoculated in TMM to grow for 18 hours. The OD_600_ was adjusted to 0.1, and 75 μL of the suspension was added to the final volume of 150 μL with copper solution (0, 5, 10, 15, 20, 25, 30, 40, 50, 75, 125 μg mL^-1^) in 96-well microplate. The initial absorbance was read in a microplate reader (Multiskan Go, Thermo Fisher, USA). For the determination of the bacterial growth, the microplate was kept on shaking in an incubator at 25°C for 24 hours, with a reading at intervals of 1h. The experiment was replicated three times. Bacterial growth curves were established to determine the minimal inhibitory concentration (MIC) at 24 hours, maximum specific growth rate (μmax), and the IC_50_ for all Psa isolates from Portugal (22) and the reference strain of Psa biovar 3 (CFBP 7286). Statistical analysis was carried out using one-way ANOVA with significant results for p<0.05 in GraphPad Prism 7.

## Results

### MLSA genotyping of Portuguese Psa isolates

**Fig 1:**
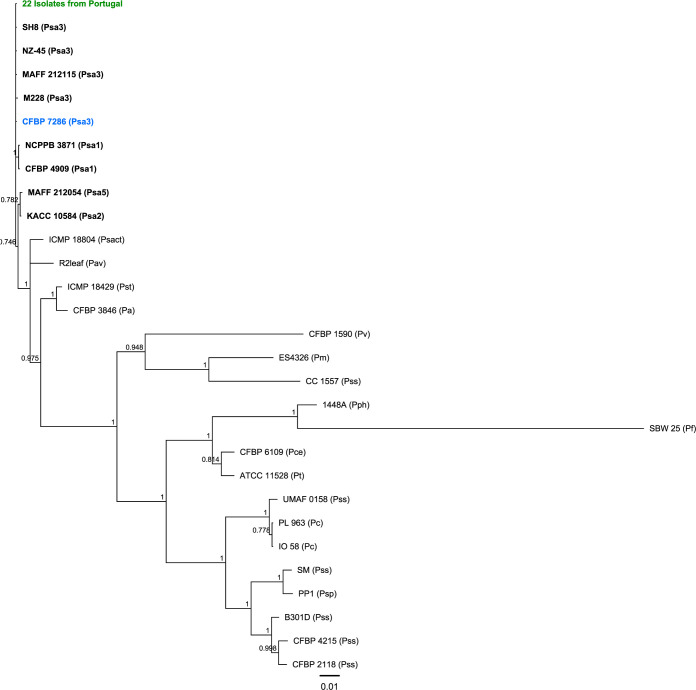
**Phylogenetic tree using four concatenated housekeeping genes (*gapA*, *gltA*, *gyrB*, and *rpoD*) with 1,950 bp from Portuguese Psa isolates between 2013 to 2017 (labeled with green codes) and Psa biovar 3 reference strain—CFBP 7286 (blue) also sequenced in this work.** Strains of Psa biovar 3 are labeled in bold characters. All bacteria used to build the dendrogram are available in S1 and S5 Tables. Bayesian tree with 1,100,000 chain length, 200 subsample frequency, a burn-in length of 110,000 and a total samples analyzed of 4,951 was used to construct the phylogenetic tree on Geneious Prime software, scale bar represents the number of the nucleotide substitutions per site. Species and pathovars are identified by abbreviation close to the strain names: *P*.*s*. pv. *actinidiae* (Psa, each biovar represented by following numbers: 1, 2, 3, 5 and 6); *P*. *viridiflava* (Pv); *P*.*s*. pv. *syringae* (Pss); *P*.*s*. pv. *maculicola* (Pm); *P*.*s*. pv. *avellanae* (Pav); *P*.*s*. pv. *actinidifoliorum* (Psact); *P*.*s*. pv. *tomato* (Pst); *P*.*s*. pv. *avii* (Pa); *P*. *cerasi* (Pc); *P*.*s*. pv. *pisi* (Pps); *P*.*s*. pv. *cerasicola* (Pce); *P*.*s*. pv. *tabaci* (Pt); *P*. *savastanoi* pv. *phaseolicola* (Pph); *P*. *fluorescens* (Pf).

Twenty-two Psa isolates from Portuguese kiwifruit orchards were identified by duplex-PCR. The molecular analysis (MLSA) using 4 housekeeping genes on these 22 isolates herewith other 28 phytopathogens *Pseudomonas* spp. (S1 and S5 Tables) reveals the consistent identification as Psa, of all isolates, from the Portuguese kiwifruit orchards. No polymorphisms were found among the 22 concatenated sequences of Portuguese isolates used for MLSA, nor with the strain CFBP 7286 (Fig 1). These data show a high clonal population structure for these Psa isolates (S1 Table) and suggest that they belong to the Psa biovar 3, recognized as the most virulent biovar and the only one so far reported to be present in Portugal [15, 18, 19, 45]. Among the 1,950 bp used to discriminate Psa3, only the M228 strain (non-pandemic Chinese Psa3 strain) showed a single polymorphism in the *rpoD* gene. MLSA allows the discrimination of Psa1, Psa2, and Psa5 biovars in different clusters. The most related P. syringe subspecies to Psa was the *P*. *syringae* pv. *avellanae* (R2leaf) with more similarity than pathogenic bacteria for *Actinidia* spp. viz. *P*. *syringae* pv. *syringae* [55], *P*. *viridiflava* [56], and *P*. *syringae* pv. *actinidifoliorum* [57] (Fig 1). The high genetic diversity within the different strains of *P*. *syringae* pv. *syringae*, isolated from different hosts (S5 Table), is demonstrated by the distribution of these strains among different clusters (Fig 1). The phytopathogen *P*. *syringae* pv. *phaseolicola* and the non-pathogen *P*. *fluorescens* were the more distant species among the 50 strains studied here (Fig 1, S1 and S5 Tables).

### Biolog analysis for phenotype charcterization in context of *P*. *syringae* complex

**Fig 2:**
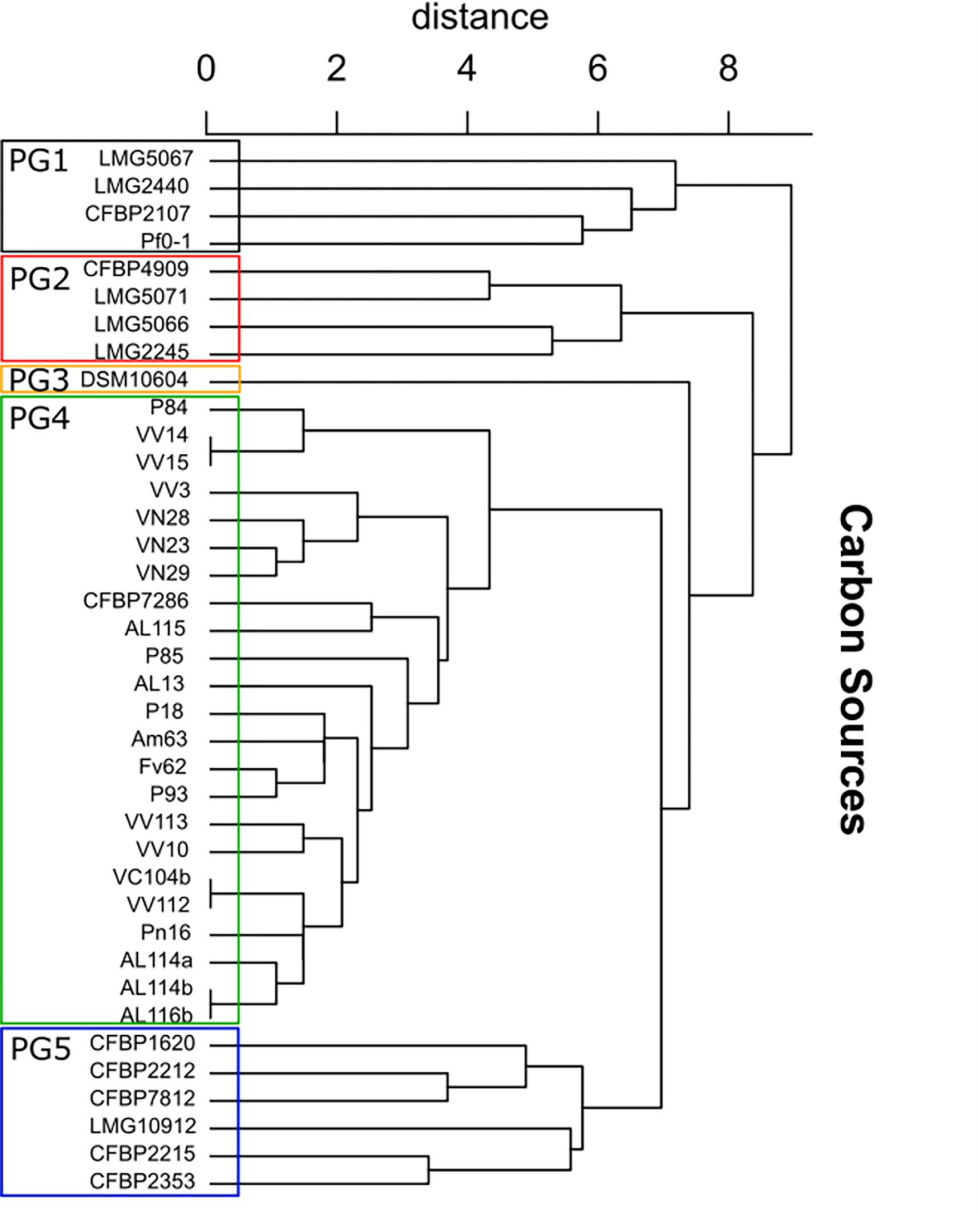
Phenotypic diversity between *Pseudomonas* spp. for the use of different carbon sources. Phenotypic groups (PG1-5) are limited by a square. The dendrogram was built from a PCA (S2 Fig) of Biolog pattern for 71 carbon sources (S7 Table). Type and reference strains used to build the dendrogram are available in S6 Table. CFBP7286 and CFB4909 are the Psa reference and type strains, identified as Psa3 and Psa1, respectively.

**Fig 3:**
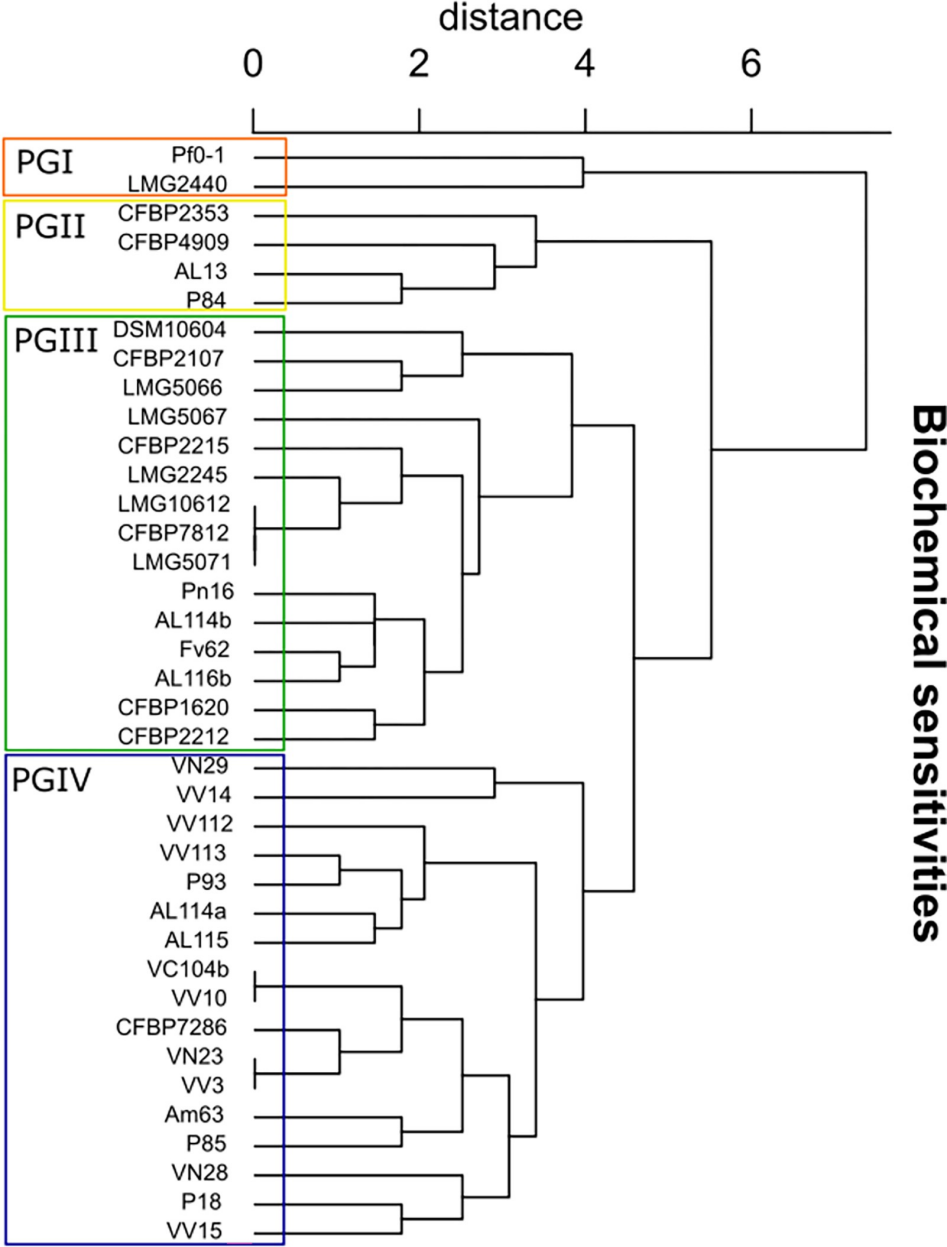
Biochemical sensitivities of different *Pseudomonas* spp. Phenotypic groups (PGI-IV) are divided into different clusters and limited by a square. The dendrogram was built from a PCA (S3 Fig) of the Biolog pattern for 24 parameters (S8 Table). The type and reference strains used are available in S6 Table. CFBP7286 and CFB4909 are the Psa reference and type strains, identified as Psa3 and Psa1, respectively.

The Biolog GENIII MicroPlate^TM^ (Biolog, 2008; Biolog, 2011) provided a biochemical characterization of the Portuguese Psa isolates in the context of the *P*. *syringae* complex. For carbon sources, a Principal Component Analysis (PCA; S2 Fig) unveiled different groups (S6 Table). All the 22 Psa isolates showed similar behavior, allocating in the PG4 cluster, which also integrated the Psa3 reference strain (Fig 2). Regarding the use of carbon sources, Psa1 (CFBP 4909) differed from Psa3 and had a profile similar to those of *P*. *savastanoi* pv. *phaseolicola*, *P*. *syringae* pv. *maculicola* and *P*. *savastanoi* pv. *glycinea*, appearing in the phenotypic group PG2 (Fig 2). Despite data showing that the 22 Psa isolates belong to the biovar 3, the main group PG4 is divided into two subgroups. One of them is constituted by CFBP 7286 and most of the Psa isolates, while another group is constituted by P84, VV14, VV15, VV3, VN28, VN23, and VN29 (Fig 3). The main differences in carbon use are reported in the S7 Table. The isolates VV3 and AL115 are the only isolates that cannot metabolize D-Mannitol and Pectin, respectively (S7 Table), the P85 metabolizes L-Galactonic Acid. Contrary to the 22 isolates of Psa used here, the isolates CFBP 7286 and CFBP 4909 cannot metabolize Glycyl-L-Proline. While β-Hydroxy-Butyric Acid is only metabolized by P84, VV14, and VV15, Acetoacetic Acid is metabolized only by VN23, VN28, VN29, and VV3 for Psa isolates. Propionic Acid is not a carbon source used by VV14 and VV15.

The Biolog GENIII MicroPlateTM screen (for 24 biochemical parameters) of the Portuguese Psa isolates and reference/type strains provided four clusters (Fig 3, S6 Table). A PCA was performed (S3 Fig), and the hierarchical disposition of the biochemical sensitivity patterns (S8 Table) showed that the Portuguese isolates were separated into three clusters (Fig 3). The phenotypic group PGII is composed of the CFBP 4909, AL13, and P84 isolates. These last isolates were isolated in 2013 and share a biochemical profile close to Psa1. The remaining Psa isolates were allocated to PGIII and PGIV. In PGIII, four Portuguese Psa isolates (Pn16, AL114b, AL116b, and Fv62) are close to *P*. *syringae* pv. *syringae*, *P*. *viridiflava*, *P*. *syringae* pv. *helianthin*, *P*. *syringae* pv. *maculicola*, *P*. *syringae* pv. *oryzae*, *P*. *savastanoi* pv. *phaseolicola*, *P*. *savastanoi* pv. *glycinea*, *P*. *syringae* pv. *delphinii*, *P*. *syringae* pv. *antirrhinin*, *P*. *syringae* pv. *tomato* and *P*. *syringae* pv. *actinidifoliorum* (Fig 3). CFBP 7286 and *P*. *syringae* pv. *theae* (CFBP 2353) isolates are allocated with the rest of the Portuguese Psa isolates (Fig 3). The main differences in biochemical sensitivities are related to P85, P93, VV113, AL114a, VN29, VV112, and VV14, with the capacity to grow in NaCl 1% compared to other Psa Portuguese isolates and CFBP 7286. On the other hand, as observed for CFBP 4909, only AL13 and P84 are susceptible to D-Serine.

Regarding sensitivity profiles, the isolates AL13 and VN29 showed sensitivity to Tetrazolium blue, such as Psa1. VV14 isolated demonstrated capacity to grow in Minocycline subtract and P18, VN29, VV14 and VV15 are tolerant to Aztreonam antibiotic.

The most distant phenotypic group is the PGI composed of *Pectobacterium carotovorum* subsp. *carotovorum* and *P*. *fluorescens* (non-pathogenic).

### Motility and IAA production by Portuguese Psa isolates

**Fig 4:**
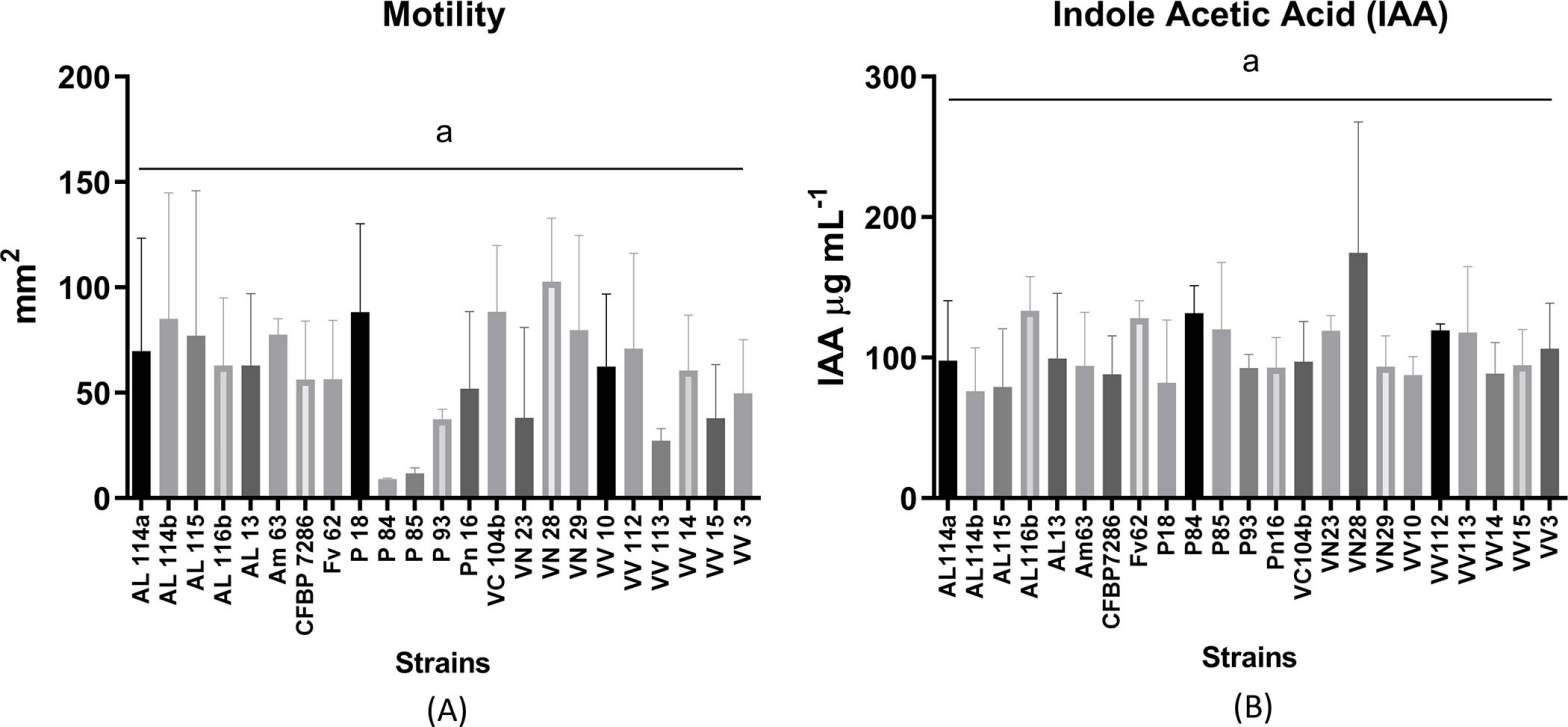
Motility performance (A) and IAA production (B) of 22 Psa isolates from Portugal, sampled between 2013 to 2017.

The analysis of 22 Portuguese Psa isolates and a Psa3 reference strain (CFBP7286) showed no significant differences in motility (Fig 4A), thus demonstrating homogeneity in this phenotypic trait. Also, the IAA production showed a similar response in all strains with no significant differences (Fig 4B), in line with the phenotypic behavior of the Psa3 reference strain.

### Psa sensitivity to copper

In general, Psa isolates are susceptible to CuSO_4_ treatments, with Minimal Inhibitory Concentration (MIC) ranging from 75 to >125 μg mL^-1^ (470 –>783μM, Table 1). From the 24h growth curves (S4 Fig), it was possible to calculate the half-maximal inhibitory concentration (IC_50_) for CuSO_4_ in each strain. Considering the IC_50_ values, AL114a was the most sensitive isolate (which is in line with MIC determination), followed by P85 and P93 (p<0.05, Table 1). The minimum CuSO_4_ concentration leading to a reduction of the maximum specific growth rate (μmax) ranged in these isolates between 10 and 40 μg mL^-1^ CuSO_4_ (Table 1). Some variability in copper sensitivity was found among the strains, with AL114a and VV10 (10 μg mL^-1^ CuSO_4_) being the most sensitives, while AL13 was the less susceptible (S9 Table). CFBP 7286 showed an in-between sensitivity corresponding to 25 μg mL^-1^ CuSO_4_ (Table 1).

**Table 1:** Copper sulfate sensitivity determination by 24h bacterial growth curves for Psa isolates from Portugal and reference strain (CFBP 7286^R^).

Strains	MIC (μg mL^-1^)	IC_50_ (μg mL^-1^)	[CuSO_4_] μmax
CFBP 7286^R^	75	41,5 ± 2,44 *b*	<25
**AL 115**	75	42,1 ± 2,80 *b*	<20
**AL 116b**	75	36,9 ± 3,22 *bc*	<25
**AL 13**	75	39,2 ± 3,25 *abc*	<40
**Pn 16**	75	37 ±2,92 *bc*	<25
**VV 112**	75	37,8 ± 2,74 *bc*	<30
**Fv 62**	75	37,6 ± 2,77 *bc*	<25
**P 93**	75	33,7 ± 4,26 c	<30
**VC 104b**	75	36,4 ± 2,39 *bc*	<15
**AL 114b**	75	37,4 ± 1,64 *bc*	<20
**P 84**	75	38,1 ± 3,08 *abc*	<25
**P 85**	75	34 ± 3,89 c	<30
**P 18**	75	38 ± 2,63 *bc*	<20
**VV 3**	75	42,8 ± 3,43 *abc*	<15
**VN 28**	75	37,2 ± 2,22 *bc*	<15
**VN 23**	75	38,7 ± 2,88 *abc*	<25
**VV 14**	75	41,3 ± 2,88 *abc*	<15
**VN 29**	75	39,7 ± 2,63 *abc*	<15
**VV 10**	75	35 ± 2,30 *bc*	<10
**VV 15**	75	38,9 ± 2,38 *abc*	<15
**Am 63**	75	43 ± 2,69 *abc*	<30
**AL114a**	>125	48 ± 5,17 *a*	<10
**VV113**	>125	44,3 ± 5,49 *ab*	<25

## Discussion

The first occurrence of Psa in Portugal was reported in 2010 [15], approximately two years before its inclusion in the EPPO list of quarantine species [12]. Following EU directives, measures took place in Portugal to combat the kiwifruit bacterial cancer [58]. Despite the efforts to adopt cultural practices towards controlling the disease impact and dispersion, Psa rapidly dispersed through all kiwifruit production areas in Portugal [59]. The incidence of this disease persists in Portugal, and the diversity of the Psa population structure has been only briefly studied. Moreover, available studies focused on small and poorly representative samples, such as covering only 1-year sampling, or few sampling places and a low number of isolates [17, 59, 60]. These studies identified a high similarity of Portuguese Psa strains with the pandemic European Psa biovar 3 lineage. The results reported here integrate 22 Psa strains isolated between 2013 to 2017 from representative kiwi-producing regions in the north of Portugal. These isolates identified as belonging to biovar Psa3 demonstrated high clonality (Fig 1) according to MLSA genotyping corroborating previous studies [18, 19, 59]. The MLSA and MLST have been broadly used for Psa genotyping [3, 17, 26, 61, 62], and these approaches were highly efficient to characterize the Psa biovars [63, 64]. The Psa biovar 3 origin has been described for China [17, 65], and distinct clonal lineages, based on China’s spread route, were found for New Zealand, Chile, and European countries [63]. Despite the geographical origin of the pandemic outbreak, the Chinese Psa3 isolates showed genomic diversity and higher similarity to the Italian strains than other European isolates, namely those found in France and Spain [66]. This finding reveals the possibility of distinct and restricted evolution episodes of Psa3 in Europe. In line with this scenario, it is crucial to characterize the Portuguese Psa collection for five years (2013–2017) and identify possible heterogeneous populations at the phenotypic level.

The phenotypic characterization contributes to understanding the bacteria’s behavior and environmental competitive advantages [67, 68], including the activity of its virulence factors [69, 70, 71]. Plant pathogenic bacteria need to move in different environments. They use flagella for motility, spreading on different surfaces, infecting the different plant structures, and moving in water [72]. When its motility is suppressed or reduced, the virulence of the pathogen is drastically reduced [38, 73]. Zhang et al. [74] demonstrated for Psa, that a minor ability for biofilm formation is reflected in a decrease in bacterial motility and a decrease in virulence. The Portuguese Psa collection studied here presented a similar *in vitro* motility performance (Fig 4A). The Chilean Psa collection showed significant differences in strains’ motility [26] and possible heterogenicity in environmental behaviour and host infection. *Pseudomonas syringae* isolates produce auxins like IAA, a phytohormone group important in bacterial pathogenicity [75]. They also produce effector proteins that control the specific mechanisms of IAA action, such as suppressing the host defences of the salicylic acid-mediated response [76–78]. The synthesized IAA plays a role during the reprogramming transcription of Psa infection in *A*. *chinesis*, which confirmed its role as a pathogenicity signaling molecule in Psa infection [78]. Our study demonstrate that there are no significant differences between Psa strains from Portugal. While the Chilean strains showed a significant diversity in the amounts of IAA produced [26]. Using the same detection method, the average values for IAA concentration after 24 hours post-inoculation ranged between ~ 20 and 60 μg mL^-1^ in the Chilean strains, while the strains collected in Portugal showed values higher than 80 μg mL^-1^ (Fig 4B). These differences regarding IAA synthesis between different populations deserve further attention.

The Biolog allows a phenomics approach and has been accurately used to understand the metabolic pathways [79] by providing a “microarray” of 71 carbon sources and 23 physiologic tests (S6 and S7 Tables). In the Pseudomonads complex group, the Biolog can discriminate the Pseudomonas spp. by the phenome profile [80, 81]. Different plant pathogen P. syringae strains have been identified by the Biolog GEN III system [7, 82–84] as well as Psa [26]. This method characterized the Psa collection (Figs 3, 4) likewise in other studies [26, 43]. For the carbon sources usage in Pseudomonad species complex, Psa3 was allocated in PG4 clade (Fig 2) but Psa biovar 1 showed a fingerprinting close to *P*. *savastanoi* pv. *phaseolicola* and *P*. *savastanoi* pv. *glycinea* (PG5, Fig 2). Differences in carbon metabolism were evidenced within the PG4 isolates, with P85 using L- Galactomic Acid. Also, VV3 and AL115 did not use D-Mannitol and Pectin. Additionally, the Portuguese strains differed from the reference and type strains (CFBP 7286 and CFBP4909) regarding the consumption of the Glycyl-L-Proline. These results show heterogeneity of the phenome of Psa strains not only within the Portuguese collection but also compared to the Chilean population. For example, two Chilean strains were not able to use D-fructose [26], contrary to all the strains isolated in Portugal that metabolized this carbon source. Also, while all Portuguese strains metabolized the Methyl Pyruvate and L-Glutamic Acid, these compounds were not metabolised by several Chilean strains [26]. These different metabolic routes reveal variability within each population and between the Portuguese and Chilean populations.

The biochemical sensitivity level of the Portuguese Psa strains demonstrated resistance to some antibiotics similar to some Chilean strains which were reported to be resistant to antibiotics Rifamycin SV and Vancomycin [26]. On the other hand, while all Chilean strains showed resistance to Lincomycin, only the Portuguese VV113 presented the same resistance. Also, all Chilean strains were susceptible to D-Serine, but only two Portuguese isolates showed the same sensitivity. The PGII and PGIV clusters, which contain the Portuguese Psa3, are separated (Fig 3) showing a high heterogenic profile in biochemical sensitivities, suggesting a differential gene expression and a possible genetic diversity unnoticed by MLSA. Ultimately, these results indicate different environmental adaptation solutions for the Portuguese biovars.

With the increasing reports of Psa isolates showing copper-resistance [41, 43], it becomes relevant to understand how Portuguese isolates tolerate copper, as most products used against Psa are based on copper solutions. The Portuguese Psa isolates did not show resistance to CuSO_4_ (concentrations up to 0.125 mM) and many of the isolates showed a MIC value around 0.075 mM, which is identical to the value of Chilean strains showing no resistance to copper [26]. The isolates AL114a and VV113 demonstrated a MIC value higher than 0.125 mM, but the IC_50_ value and μmax are in line with the values of the other isolates (Table 1). The MIC at around 0.125 mM of CuSO_4_ is so far from the copper resistance values found in Psa between 1.8 to 2.4 mM of CuSO_4_ in Japan [44] and 1.2 mM in New Zealand [41].

Although these phenotypic profiles unveil different clusters for Psa strains isolated in Portugal (Figs 2 and 4), the observed genetic/phenotypic variation did not show significant differences between the localization of the Psa isolation (counties) using strains sampled between 2013–2017 (S5 Fig). These results suggest a diversified source of the Psa spreading in the north of Portugal, which indicate different outbreaks and maybe a cross spread by different counties. Also, it may suggest there is not a pattern for local adaptive Psa phenotypes/genotypes, thus Psa strains remain highly conserved comparing different orchard locations.

## Conclusions

In conclusion, the results in this study indicate that the Psa isolates from Portugal (2013–2017) belong to Psa biovar 3. No polymorphisms were observed between the concatenated sequences of the 22 Psa isolates used for (1950 bp), revealing a high clonal Psa population structure. This characterization using conventional locus has a low capacity to distinguish among Psa biovar 3 cluster, despite an SNP being found at the M228 Chinese strain. For studies focusing on the genetic characterization of Psa using locus sequencing, other approaches like whole-genome sequencing should be adopted to clarify the populational structure identity. Based on sampling, Psa strains from Portugal may have resulted from a pandemic lineage (Psa3) from the Italy outbreak in 2008. However, phenotypic differences were detected, as different Biolog patterns were observed for carbon sources and biochemical sensitivities. This heterogenicity can reveal genetic differences in the background of the metabolic pathways. However, phenotypic differences were detected, as different Biolog patterns were observed for carbon sources and biochemical metabolism and sensitivities. This heterogeneity evokes different metabolic pathways and genetic differences, that may reflect strain-specific adaptations worth to pursue by comparative genomics studies. These results allowed discriminating Psa with heterogeneous behavior, which will be used in future sequencing analysis of Next Generation Sequence. Furthermore, by demonstrating the sensitivity to Cu of these 22 Psa Portuguese isolates, this study ultimately allowed to fill a critical gap for the producers regarding the effectiveness of Cu-based treatments to control bacterial canker caused by Psa in Portugal.

## Supporting information

S1 Fig Identification of *Pseudomonas syringae pv. actinidiae* using a specific duplex-PCR. Molecular ladder GeneRuler 1 kb Plus DNA Ladder was used (M).
(DOCX)

S2 Fig Principal component analysis for carbon source usage by Pseudomonad complex in Biolog GEN III. (DOCX)

S3 Fig Principal component analysis for biochemical sensitivities in the Pseudomonad complex by Biolog GEN III. (DOCX)

S4 Fig Bacterial growth curves in Tris Minimal Medium (TMM) under different CuSO_4_ concentrations (ranged between 0 to 125 μM). (DOCX)

S5 Fig Principal component analysis (PCA) using all data obtained in this study and clustering analysis for a confidence interval of 95% by sampling origin of the Psa isolates (Amarante, Amares, Felgueiras, Penafiel, Prado, Valença, Vila do Conde and Vila Verde). (DOCX)

S1 Table Identity of the Portuguese Psa isolates. (DOCX)

S2 Table Primers used for Psa identification by duplex-PCR. (DOCX)

S3 Table Primers used for Multi-Locus Sequence Analysis. (DOCX)

S4 Table Accession number of the sequenced partial genes in GenBank at NCBI. (DOCX)

S5 Table Identification of the bacterial strains used for MLSA analysis. (DOCX)

S6 Table List of the bacterial strains used to identify the Biolog pattern for Pseudomonad complex. (DOCX)

S7 Table Carbon source pattern obtained for Portuguese Psa isolates by BIOLOG GEN III. (DOCX)

S8 Table Biochemical pattern obtained for Portuguese Psa isolates by BIOLOG GEN III. (DOCX)

S9 Table Maximum growth rate from bacteria growth curves under different doses of copper. The bold numbers and (*) mean significant differences to control (0 CuSO_4_) according to p<0.05.
(DOCX)
