# Supplementary material for: Distinct phenotypic behaviours within a clonal population of Pseudomonas syringae pv. actinidiae
Source: PLoS One. 2022 Jun 9;17(6):e0269343. doi: 10.1371/journal.pone.0269343 (PMC9182710; doi:10.1371/journal.pone.0269343)
Supplement: S1 Fig — Molecular ladder GeneRuler 1 kb Plus DNA Ladder was used (M). (DOCX) [file pone.0269343.s001.docx]

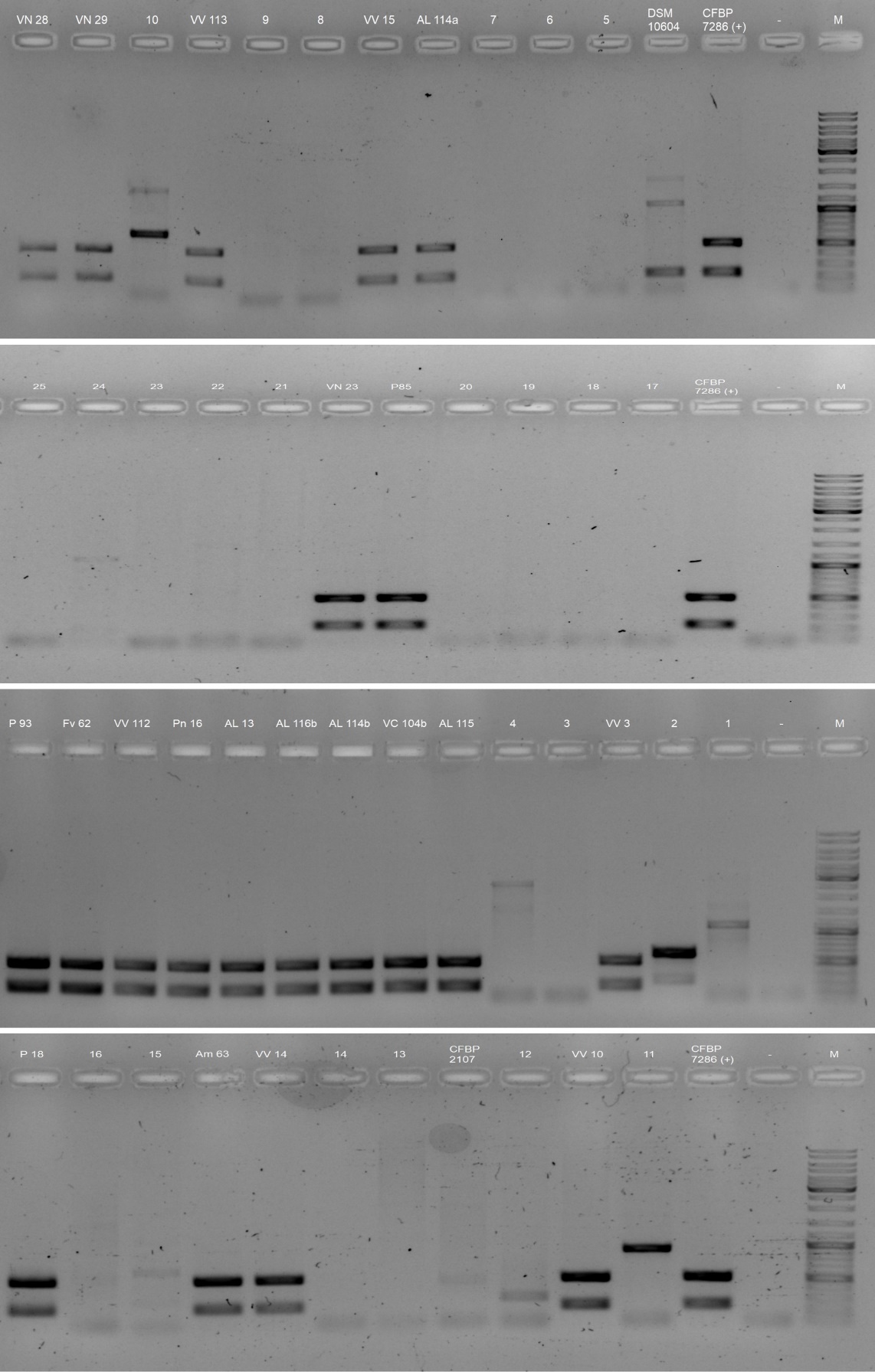


***Figure S1 –*** *Identification of Pseudomonas syringae pv. actinidiae using a specific duplex-PCR. Molecular ladder GeneRuler 1 kb Plus DNA Ladder was used (M).*
