# Supplementary material for: Distinct phenotypic behaviours within a clonal population of Pseudomonas syringae pv. actinidiae
Source: PLoS One. 2022 Jun 9;17(6):e0269343. doi: 10.1371/journal.pone.0269343 (PMC9182710; doi:10.1371/journal.pone.0269343)
Supplement: S3 Fig — (DOCX) [file pone.0269343.s003.docx]

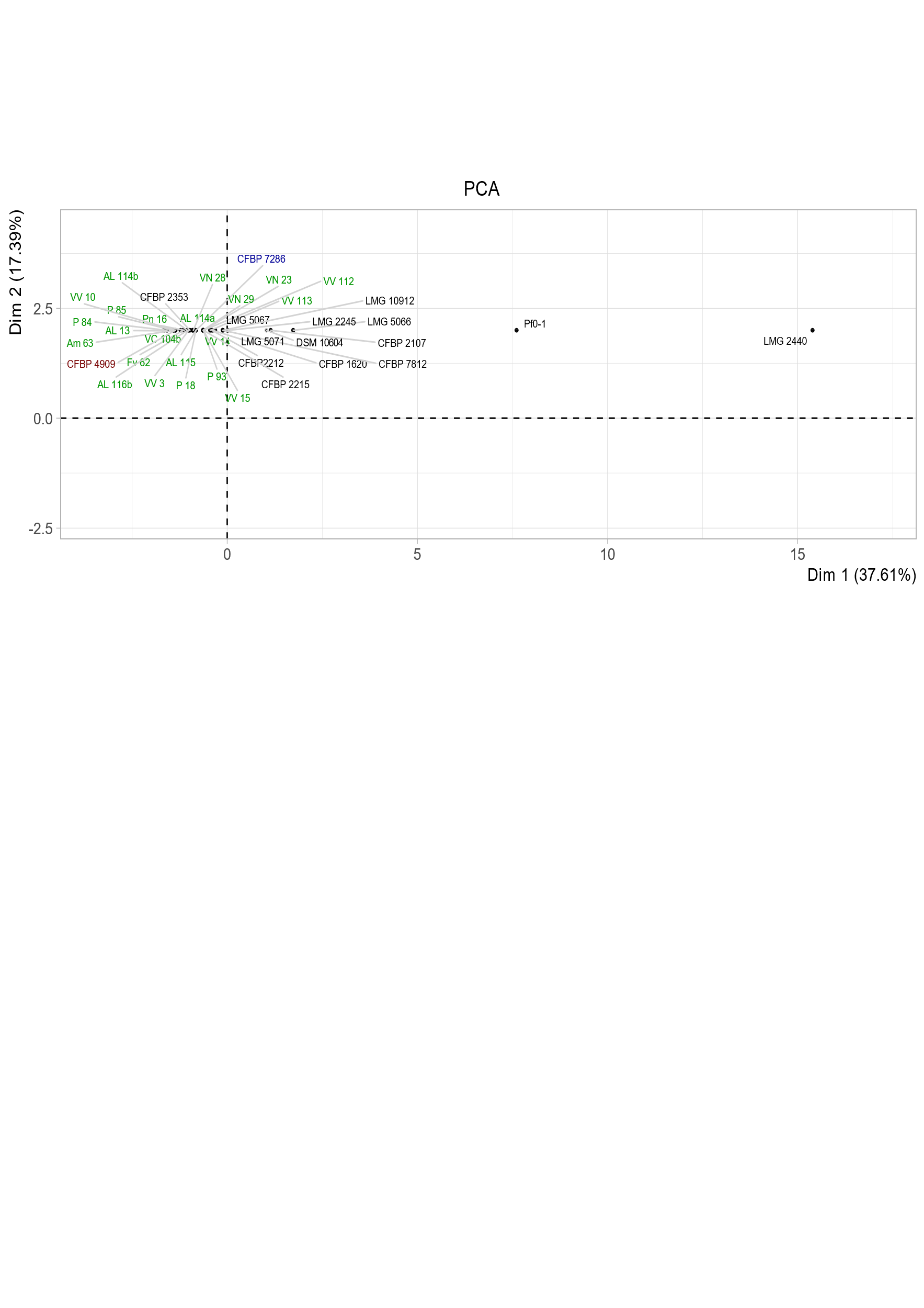


**Figure S3 -** *Principal Component analysis for biochemical sensitivities in the Pseudomonad complex by Biolog GEN III.*
