# Supplementary material for: Distinct phenotypic behaviours within a clonal population of Pseudomonas syringae pv. actinidiae
Source: PLoS One. 2022 Jun 9;17(6):e0269343. doi: 10.1371/journal.pone.0269343 (PMC9182710; doi:10.1371/journal.pone.0269343)
Supplement: S4 Fig — (DOCX) [file pone.0269343.s004.docx]

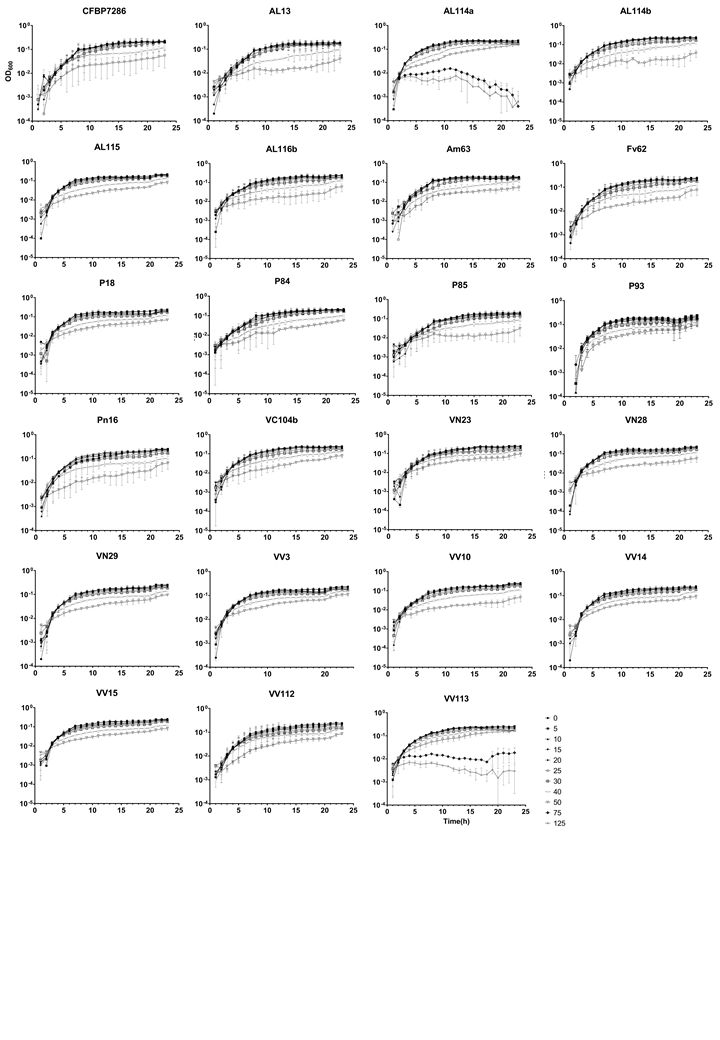


**Figure S4 –** Bacterial growth curves in Tris Minimal Medium (TMM) under different CuSO_4_ concentrations (ranged between 0 to 125 µM).
