# Supplementary material for: Distinct phenotypic behaviours within a clonal population of Pseudomonas syringae pv. actinidiae
Source: PLoS One. 2022 Jun 9;17(6):e0269343. doi: 10.1371/journal.pone.0269343 (PMC9182710; doi:10.1371/journal.pone.0269343)
Supplement: S5 Fig — (DOCX) [file pone.0269343.s005.docx]

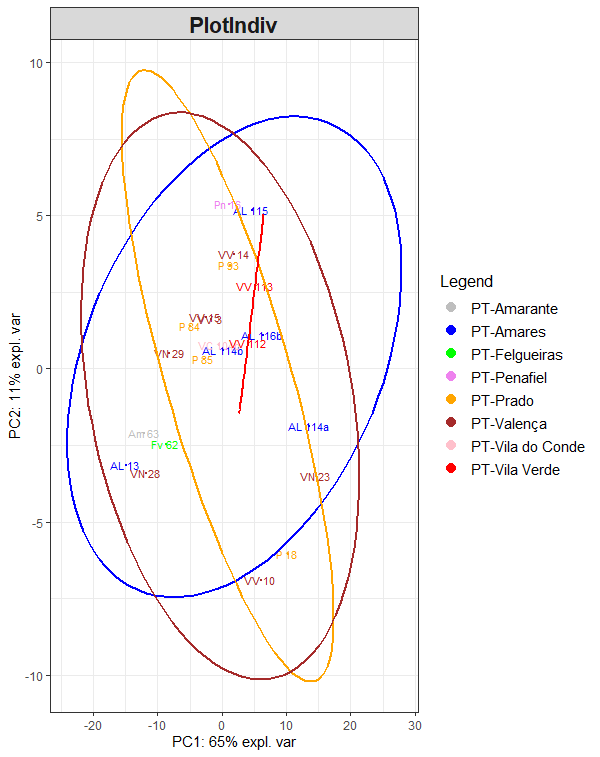


**Figure S5 –** Principal component analysis (PCA) using all data obtained in this study and clustering analysis for a confidence interval of 95% by sampling origin of the Psa isolates (Amarante, Amares, Felgueiras, Penafiel, Prado, Valença, Vila do Conde and Vila Verde).
