# Supplementary material for: Distinct phenotypic behaviours within a clonal population of Pseudomonas syringae pv. actinidiae
Source: PLoS One. 2022 Jun 9;17(6):e0269343. doi: 10.1371/journal.pone.0269343 (PMC9182710; doi:10.1371/journal.pone.0269343)
Supplement: S1 Table — (DOCX) [file pone.0269343.s006.docx]

**Table S1 –** Identity of Psa strains isolated in Portugal.

| Isolate | Host Plant | Cultivar | Origin Location | Year of Isolation | Plant material |
| --- | --- | --- | --- | --- | --- |
| AL114a | *Actinidia deliciosa* | Chieftain (♂) | PT - Amares | 2014 | Leaf |
| AL114b | *A. deliciosa* | Chieftain (♂) | PT - Amares | 2014 | Leaf |
| AL115 | *A. deliciosa* | Tsechelidis (♀) | PT - Amares | 2014 | Leaf |
| AL116b | *A. deliciosa* | Tsechelidis (♀) | PT - Amares | 2014 | Leaf |
| AL13 | *A. deliciosa* | Tsechelidis (♀) | PT - Amares | 2013 | Leaf |
| Am63 | *A. deliciosa* | n.d. | PT – Amarante | 2013 | Leaf |
| Fv62 | *A. deliciosa* | Matua (♂) | PT - Felgueiras | 2013 | Leaf |
| P18 | *A. deliciosa* | n.d. | PT - Prado | 2013 | Stem |
| P84 | *A. deliciosa* | n.d. | PT - Prado | 2013 | Leaf |
| P85 | *A. deliciosa* | n.d. | PT - Prado | 2013 | Leaf |
| P93 | *A. deliciosa* | n.d. | PT - Prado | 2013 | Leaf |
| Pn16 | *A. deliciosa* | Hayward (♀) | PT - Penafiel | 2013 | Flower buds |
| VC104b | *A. deliciosa* | Hayward (♀) | PT - Vila do Conde | 2013 | Leaf |
| VN23 | *A. deliciosa* | Hayward (♀) | PT - Valença | 2016 | Leaf |
| VN28 | *A. deliciosa* | Hayward (♀) | PT - Valença | 2017 | Flower |
| VN29 | *A. deliciosa* | Hayward (♀) | PT - Valença | 2017 | - |
| VV3 | *A. deliciosa* | Hayward (♀) | PT - Valença | 2016 | Leaf |
| VV10 | *A. deliciosa* | n.d. (♂) | PT - Valença | 2017 | Leaf |
| VV14 | *A. deliciosa* | Hayward (♀) | PT - Valença | 2017 | Flower |
| VV15 | *A. deliciosa* | Hayward (♀) | PT - Valença | 2017 | Bacterial ooze |
| VV112 | *A. deliciosa* | Hayward (♀) | PT - Vila Verde | 2014 | Leaf |
| VV113 | *A. deliciosa* | Tomuri (♂) | PT - Vila Verde | 2014 | Leaf |
