# Supplementary material for: Distinct phenotypic behaviours within a clonal population of Pseudomonas syringae pv. actinidiae
Source: PLoS One. 2022 Jun 9;17(6):e0269343. doi: 10.1371/journal.pone.0269343 (PMC9182710; doi:10.1371/journal.pone.0269343)
Supplement: S2 Table — (DOCX) [file pone.0269343.s007.docx]

**Table S2 –** Primers used for Psa identification by Duplex-PCR.

| PCR | Primers | Primer Sequence | Size (bp) | Reference | Guideline |
| --- | --- | --- | --- | --- | --- |
| Duplex-PCR | AvrDdpx - F | 5’ TTTCGGTGGTAACGTTGGCA 3’ | 226 | Gallelli *et al*., 2011 | EPPO, 2014 |
|  | AvrDdpx - R | 5’ TTCCGCTAGGTGAAAAATGGG 3’ |  |  |  |
|  | KN - F | 5’ CACGATACATGGGCTTATGC 3’ | 492 | Koh and Nou, 2002 |  |
|  | KN - R | 5’ CTTTTCATCCACACACTCCG 3’ |  |  |  |
