# Supplementary material for: Distinct phenotypic behaviours within a clonal population of Pseudomonas syringae pv. actinidiae
Source: PLoS One. 2022 Jun 9;17(6):e0269343. doi: 10.1371/journal.pone.0269343 (PMC9182710; doi:10.1371/journal.pone.0269343)
Supplement: S3 Table — (DOCX) [file pone.0269343.s008.docx]

**Table S3 –** Primers used for Multi-Locus Sequence Analysis.

| PCR | Primers | Primer Sequence | Annealing (ºC) | Reference |
| --- | --- | --- | --- | --- |
| Multi-Locus Sequence Analysis (MLSA) | gapA-F | 5’ CGCCATYCGCAACCCG 3’ | 60 | Sarkar and Guttman, (2004) |
|  | gapA-R | 5’ CCCAYTCGTTGTCGTACCA 3’ |  |  |
|  | gltA-F | 5’ AGTTGATCATCGAGGGCGCWGCC 3’ | 60 |  |
|  | gltA-R | 5’ TGATCGGTTTGATCTCGCACGG 3’ |  |  |
|  | gyrB-F | 5’ MGGCGGYAAGTTCGATGACAAYTC 3’ | 60 |  |
|  | gyrB-R | 5’ TRATBKCAGTCARACCTTCRCGSGC 3’ |  |  |
|  | rpoD-F | 5’ AAGGCGARATCGAAATCGCCAAGCG 3’ | 65 |  |
|  | rpoD-R | 5’ GGAACWKGCGCAGGAGTCGGCACG 3’ |  |  |

`
