# Supplementary material for: Distinct phenotypic behaviours within a clonal population of Pseudomonas syringae pv. actinidiae
Source: PLoS One. 2022 Jun 9;17(6):e0269343. doi: 10.1371/journal.pone.0269343 (PMC9182710; doi:10.1371/journal.pone.0269343)
Supplement: S4 Table — (DOCX) [file pone.0269343.s009.docx]

**Table S4 –** Accession Number of the sequenced partial genes in GenBank at NCBI.

|  | **NCBI Accession Number** | | | |
| --- | --- | --- | --- | --- |
| **Strain** | ***gapA*** | ***gltA*** | ***gyrB*** | ***rpoD*** |
| AL13 | MT812509 | MT812510 | MT812511 | MT812512 |
| AL114a | MT812513 | MT812514 | MT812515 | MT812516 |
| AL114b | MT812517 | MT812518 | MT812519 | MT812520 |
| AL115 | MT812521 | MT812522 | MT812523 | MT812524 |
| AL116b | MT812525 | MT812526 | MT812527 | MT812528 |
| Am63 | MT812529 | MT812530 | MT812531 | MT812532 |
| Fv62 | MT812533 | MT812534 | MT812535 | MT812536 |
| P18 | MT812537 | MT812538 | MT812539 | MT812540 |
| P84 | MT812541 | MT812542 | MT812543 | MT812544 |
| P85 | MT812545 | MT812546 | MT812547 | MT812548 |
| P93 | MT812549 | MT812550 | MT812551 | MT812552 |
| Pn16 | MT812553 | MT812554 | MT812555 | MT812556 |
| VC104b | MT812557 | MT812558 | MT812559 | MT812560 |
| VN23 | MT812561 | MT812562 | MT812563 | MT812564 |
| VN28 | MT812565 | MT812566 | MT812567 | MT812568 |
| VN29 | MT812569 | MT812570 | MT812571 | MT812572 |
| VV3 | MT812573 | MT812574 | MT812575 | MT812576 |
| VV10 | MT812577 | MT812578 | MT812579 | MT812580 |
| VV14 | MT812581 | MT812582 | MT812583 | MT812584 |
| VV15 | MT812585 | MT812586 | MT812587 | MT812588 |
| VV112 | MT812589 | MT812590 | MT812591 | MT812592 |
| VV113 | MT812593 | MT812594 | MT812595 | MT812596 |
| CFBP 7286* | - | - | - | - |
