# Supplementary material for: Distinct phenotypic behaviours within a clonal population of Pseudomonas syringae pv. actinidiae
Source: PLoS One. 2022 Jun 9;17(6):e0269343. doi: 10.1371/journal.pone.0269343 (PMC9182710; doi:10.1371/journal.pone.0269343)
Supplement: S5 Table — (DOCX) [file pone.0269343.s010.docx]

**Table S5 –** Identification of the bacterial strains used for MLSA analysis.

| Strain | Specie | Country | Isolation Year | Host (source) | NCBI BioSample Accession |
| --- | --- | --- | --- | --- | --- |
| CFBP 7286 | *Pseudomonas syrinage pv. Actinidiae* (biovar 3) | Italy | 2008 | *Actinidia deliciosa* | SAMN02471738 |
| MAFF 212115 | *P.s. pv. actinidiae* (Biovar 3) | Japan | 2014 | *Solanum lycopersicum* | SAMN08031203 |
| Shaanxi_M228 | *P.s. pv. actinidiae* (Biovar 3) | China | 2010 | *A. deliciosa* | SAMN02471925 |
| KACC 10584 | *P.s. pv. actinidiae* (Biovar 2) | South Korea | 1997 | *A. chinensis* | - |
| SH8 | *P.s. pv. actinidiae* (Biovar 3) | China | 2013 | *A. chinensis* | - |
| ICMP 20586 | *P.s. pv. actinidiae* (Biovar 3) | New Zealand | 2014 | *A. deliciosa* | SAMN05589874 |
| CFBP 4909^T^ | *P.s. pv. actinidiae* (Biovar 1) | Japan | 1984 | *A. deliciosa* | SAMN02740942 |
| NCPPB 3871 | *P.s. pv. actinidiae* (Biovar 1) | Italy | 1992 | *A. deliciosa* | SAMN02471301 |
| MAFF 212054 | *P.s. pv. actinidiae* (Biovar 5) | Japan | 2012 | *A. chinensis* | - |
| MAFF 212140 | *P.s. pv. actinidiae* (Biovar 6) | Japan | 2015 | *A. deliciosa* | - |
| ICMP 18804 | *P.s. pv. actinidifoliorum* | New Zealand | 2010 | *A. chinensis* | SAMN02471913 |
| B301D | *P.s. pv. syringae* | United Kingdom | 1959 | *Pyrus communis* | SAMN03267739 |
| CFBP 2118 | *P.s. pv. syringae* | France | 1979 | *Prunus cerasus* | SAMEA104365386 |
| CFBP 4215 | *P.s. pv. syringae* | France | 1997 | *P. avium* | SAMEA104365387 |
| SM | *P.s. pv. syringae* | USA | - | *Triticum aestivum* | SAMN02472030 |
| UMAF 0158 | *P.s. pv. syringae* | Spain | 1992-1997 | *Mangifera indica* | SAMN04053740 |
| CC 1557 | *P.s. pv. syringae* | France | - | snow | SAMN02471566 |
| CFBP 1590 | *P. viridiflava* | France | 1974 | *P. cerasus* | SAMEA104090973 |
| IO58^T^ | *P. cerasi* | Poland | 2007 | *P. cerasus* | - |
| PL963 | *P. cerasi* | Poland | 2009 | *P. avium* | - |
| SBW25 | *P. fluorescens* | United Kingdom | 1989 | *Beta vulgaris* | SAMEA2272316 |
| CFBP 6109 | *P.s. pv. cerasicola* | Japan | 1995 | *P. yedoensis* | GCF_001537945.1 |
| 1448A | *P. savastanoi pv. phaseolicola* | - | - | *Phaseolus vulgaris* | SAMN02603162 |
| ICMP 3078 | *P.s. pv. pisi* | Japan | 1951 | *Pisum sativum* | SAMN02471574 |
| ATCC 11528 | *P.s. pv. tabaci* | - | - | *Nicotiana tabacum* | SAMN00002878 |
| CFBP 3846 | *P.s. pv. avii* | France | 1991 | *P. avium* | SAMN03992187 |
| ES4326 | *P.s. pv. maculicola* | Canada | - | *Raphanus sativus* | SAMN02471321 |
| CMP 18429^T^ | *P.s. pv. tomato* | Guernsey | 1960 | *S. lycopersicum* | SAMN02604017 |
| R2leaf | *P.s. avellanae* | United Kingdom | 2014 | *P. avium* | SAMN05861164 |
