# Supplementary material for: Distinct phenotypic behaviours within a clonal population of Pseudomonas syringae pv. actinidiae
Source: PLoS One. 2022 Jun 9;17(6):e0269343. doi: 10.1371/journal.pone.0269343 (PMC9182710; doi:10.1371/journal.pone.0269343)
Supplement: S6 Table — (DOCX) [file pone.0269343.s011.docx]

**Table S6 -** List of the bacterial strains used to identify the Biolog pattern for Pseudomonad complex.

| Strain | Specie | Country | Isolation Year | Host (source) |
| --- | --- | --- | --- | --- |
| CFBP 7286^r^ | *Pseudomonas syrinage pv. Actinidiae* (biovar 3) | Italy | 2008 | *Actinidia deliciosa* |
| CFBP 4909^T^ | *P.s. pv. actinidiae* (biovar 1) | Japan | 1984 | *A. deliciosa* |
| LMG 2440^T^ | *Pectobacterium carotovorum subsp. carotovorum* | Italy | 1973 | *Foeniculum vulgare* |
|  |  |  |  |  |
| LMG 5067^T^ | *P.s. pv. helianthi* | Mexico | 1974 | *Helianthus annuus* |
| LMG 5071^T^ | *P.s. pv. maculicola* | New Zealand | 1965 | *Brassica oleracea* |
| LMG 10912^T^ | *P.s. pv. oryzae* | Japan | 1983 | *Oryza sativa* |
| LMG 2245^T^ | *P. savastanoi pv. phaseolicola* | Canada | 1945 | *Phaseolus vulgaris* |
| LMG 5066^T^ | *P. savastanoi pv. glycinea* | New Zealand | 1968 | *Glycine max* |
| DSM 10604^T^ | *P.s. pv. syringae* | United Kingdom | 1996 | *Syringa vulgaris* |
| Pf0-1 | *P. fluorescens* | - | - | (Soil) |
| CFBP 2107^T^ | *P. viridiflava* | Switzerland | 1927 | *Phaseolus sp.* |
| cfbp 2353^t^ | *P.s. pv. theae* | Japan | 1970 | *Camellia sinensis* |
| cfbp2215^t^ | *P.s. pv. delphinii* | New Zealand | 1957 | *Delphinium sp.* |
| cfbp 1620^t^ | *P.s. pv. antirrhini* | United Kingdom | 1965 | *Antirrhinum majus* |
| CFBP 7812 | *P.s. pv. actinidifoliorum* | New Zealand | 2010 | *A. chinensis* |
| cfbp 2212^t^ | *P.s. pv. tomato* | United Kingdom | 1960 | *Solanum lycopersicum* |
