# Supplementary material for: Distinct phenotypic behaviours within a clonal population of Pseudomonas syringae pv. actinidiae
Source: PLoS One. 2022 Jun 9;17(6):e0269343. doi: 10.1371/journal.pone.0269343 (PMC9182710; doi:10.1371/journal.pone.0269343)
Supplement: S9 Table — The bold numbers and (*) mean significant differences to control (0 CuSO4) according to p<0.05. (DOCX) [file pone.0269343.s014.docx]

**Table S9 –** Maximum growth rate from bacteria growth curves under different doses of copper. The bold numbers and (*) means significant differences to control (0 CuSO_4_) according p<0.05.

|  | Fv62 | |  | Pn16 | |  | VC104b | |  | AL114b | |  | CFBP7286 | |  | AL116b | |  |
| --- | --- | --- | --- | --- | --- | --- | --- | --- | --- | --- | --- | --- | --- | --- | --- | --- | --- | --- |
| [CuSO4] | *µmax (h-1)* | *sd* |  | *µmax (h-1)* | *sd* |  | *µmax (h-1)* | *sd* |  | *µmax (h-1)* | *sd* |  | *µmax (h-1)* | *sd* |  | *µmax (h-1)* | *sd* |  |
| 0 | 0,19 | 0,010 |  | 0,17 | 0,005 |  | 0,18 | 0,014 |  | 0,18 | 0,017 |  | 0,18 | 0,032 |  | 0,18 | 0,010 |  |
| 5 | 0,20 | 0,011 |  | 0,17 | 0,009 |  | 0,19 | 0,011 |  | 0,18 | 0,013 |  | 0,18 | 0,016 |  | 0,18 | 0,005 |  |
| 10 | 0,19 | 0,010 |  | 0,17 | 0,026 |  | 0,17 | 0,015 |  | 0,17 | 0,023 |  | 0,18 | 0,015 |  | 0,18 | 0,002 |  |
| 15 | 0,18 | 0,020 |  | 0,14 | 0,007 |  | **0,16** | **0,007** | * | 0,16 | 0,003 |  | 0,17 | 0,011 |  | 0,17 | 0,016 |  |
| 20 | 0,17 | 0,016 |  | 0,14 | 0,014 |  | **0,16** | **0,017** | * | **0,15** | **0,005** | * | 0,16 | 0,018 |  | 0,16 | 0,018 |  |
| 25 | **0,14** | **0,012** | * | **0,12** | **0,007** | * | **0,13** | **0,003** | * | **0,13** | **0,007** | * | **0,13** | **0,016** | * | **0,13** | **0,010** | * |
| 30 | **0,11** | **0,011** | * | **0,09** | **0,022** | * | **0,11** | **0,011** | * | **0,10** | **0,001** | * | **0,11** | **0,015** | * | **0,11** | **0,011** | * |
| 40 | **0,06** | **0,005** | * | **0,06** | **0,014** | * | **0,07** | **0,004** | * | **0,05** | **0,005** | * | **0,05** | **0,011** | * | **0,05** | **0,009** | * |
| 50 | **0,02** | **0,008** | * | **0,02** | **0,005** | * | **0,02** | **0,006** | * | **0,02** | **0,011** | * | **0,02** | **0,024** | * | **0,02** | **0,004** | * |
| 75 | **0,00** | **0,000** | * | **0,00** | **0,000** | * | **0,00** | **0,000** | * | **0,00** | **0,000** | * | **0,00** | **0,000** | * | **0,00** | **0,000** | * |
| 125 | **0,00** | **0,000** | * | **0,00** | **0,000** | * | **0,00** | **0,000** | * | **0,00** | **0,000** | * | **0,00** | **0,000** | * | **0,00** | **0,000** | * |
|  |  |  |  |  |  |  |  |  |  |  |  |  |  |  |  |  |  |  |
|  | VN23 | |  | VV112 | |  | P93 | |  | P84 | |  | P85 | |  | AL13 | |  |
| [CuSO4] | *µmax (h-1)* | *sd* |  | *µmax (h-1)* | *sd* |  | *µmax (h-1)* | *sd* |  | *µmax (h-1)* | *sd* |  | *µmax (h-1)* | *sd* |  | *µmax (h-1)* | *sd* |  |
| 0 | 0,19 | 0,037 |  | 0,17 | 0,036 |  | 0,19 | 0,014 |  | 0,15 | 0,028 |  | 0,13 | 0,014 |  | 0,15 | 0,017 |  |
| 5 | 0,19 | 0,019 |  | 0,17 | 0,025 |  | 0,18 | 0,012 |  | 0,14 | 0,028 |  | 0,14 | 0,007 |  | 0,15 | 0,026 |  |
| 10 | 0,19 | 0,038 |  | 0,17 | 0,038 |  | 0,19 | 0,028 |  | 0,14 | 0,014 |  | 0,13 | 0,005 |  | 0,15 | 0,034 |  |
| 15 | 0,17 | 0,025 |  | 0,15 | 0,021 |  | 0,18 | 0,029 |  | 0,13 | 0,011 |  | 0,13 | 0,004 |  | 0,15 | 0,037 |  |
| 20 | 0,16 | 0,024 |  | 0,15 | 0,026 |  | 0,19 | 0,015 |  | 0,11 | 0,023 |  | 0,12 | 0,015 |  | 0,14 | 0,028 |  |
| 25 | **0,13** | **0,017** | * | 0,12 | 0,028 |  | 0,15 | 0,011 |  | **0,09** | **0,022** | * | 0,10 | 0,006 |  | 0,12 | 0,019 |  |
| 30 | **0,10** | **0,033** | * | **0,11** | **0,039** | * | **0,13** | **0,014** | * | **0,06** | **0,030** | * | **0,08** | **0,032** | * | 0,09 | 0,003 |  |
| 40 | **0,06** | **0,027** | * | **0,30** | **0,400** | * | **0,07** | **0,012** | * | **0,04** | **0,023** | * | **0,05** | **0,010** | * | **0,05** | **0,014** | * |
| 50 | **0,03** | **0,019** | * | **0,03** | **0,012** | * | **0,04** | **0,003** | * | **0,02** | **0,018** | * | **0,02** | **0,008** | * | **0,02** | **0,010** | * |
| 75 | **0,00** | **0,000** | * | **0,00** | **0,000** | * | **0,00** | **0,000** | * | **0,00** | **0,000** | * | **0,00** | **0,000** | * | **0,00** | **0,000** | * |
| 125 | **0,00** | **0,000** | * | **0,00** | **0,000** | * | **0,00** | **0,000** | * | **0,00** | **0,000** | * | **0,00** | **0,000** | * | **0,00** | **0,000** | * |
|  |  |  |  |  |  |  |  |  |  |  |  |  |  |  |  |  |  |  |
|  | AL115 | |  | VN29 | |  | VV10 | |  | VV14 | |  | VV15 | |  | VN28 | |  |
| [CuSO4] | *µmax (h-1)* | *sd* |  | *µmax (h-1)* | *sd* |  | *µmax (h-1)* | *sd* |  |  | *sd* |  | *µmax (h-1)* | *sd* |  | *µmax (h-1)* | *sd* |  |
| 0 | 0,19 | 0,026 |  | 0,19 | 0,004 |  | 0,16 | 0,009 |  | 0,19 | 0,004 |  | 0,19 | 0,015 |  | 0,21 | 0,008 |  |
| 5 | 0,17 | 0,019 |  | 0,18 | 0,010 |  | 0,15 | 0,009 |  | 0,18 | 0,016 |  | 0,18 | 0,016 |  | 0,20 | 0,020 |  |
| 10 | 0,16 | 0,026 |  | 0,18 | 0,014 |  | **0,14** | **0,011** | * | 0,18 | 0,012 |  | 0,18 | 0,010 |  | 0,19 | 0,017 |  |
| 15 | 0,16 | 0,011 |  | **0,16** | **0,002** | * | **0,14** | **0,011** | * | **0,15** | **0,006** | * | **0,15** | **0,008** | * | **0,17** | **0,011** | * |
| 20 | **0,14** | **0,001** | * | **0,15** | **0,007** | * | **0,12** | **0,005** | * | **0,14** | **0,008** | * | **0,14** | **0,008** | * | **0,16** | **0,009** | * |
| 25 | **0,12** | **0,003** | * | **0,13** | **0,004** | * | **0,09** | **0,001** | * | **0,13** | **0,005** | * | **0,12** | **0,005** | * | **0,14** | **0,006** | * |
| 30 | **0,40** | **0,509** | * | **0,11** | **0,006** | * | **0,08** | **0,007** | * | **0,11** | **0,008** | * | **0,10** | **0,008** | * | **0,11** | **0,010** | * |
| 40 | **0,22** | **0,303** | * | **0,06** | **0,000** | * | **0,05** | **0,002** | * | **0,07** | **0,007** | * | **0,06** | **0,004** | * | **0,06** | **0,005** | * |
| 50 | **0,03** | **0,007** | * | **0,30** | **0,032** | * | **0,02** | **0,006** | * | **0,05** | **0,005** | * | **0,03** | **0,003** | * | **0,03** | **0,003** | * |
| 75 | **0,00** | **0,000** | * | **0,00** | **0,000** | * | **0,00** | **0,000** | * | **0,00** | **0,000** | * | **0,00** | **0,000** | * | **0,00** | **0,000** | * |
| 125 | **0,00** | **0,000** | * | **0,00** | **0,000** | * | **0,00** | **0,000** | * | **0,00** | **0,000** | * | **0,00** | **0,000** | * | **0,00** | **0,000** | * |
|  |  |  |  |  |  |  |  |  |  |  |  |  |  |  |  |  |  |  |
|  | VV113 | |  | AL114a | |  | Am63 | |  | VV3 | |  | P18 | |  |  |  |  |
| [CuSO4] | *µmax (h-1)* | *sd* |  | *µmax (h-1)* | *sd* |  | *µmax (h-1)* | *sd* |  | *µmax (h-1)* | *sd* |  | *µmax (h-1)* | *sd* |  |  |  |  |
| 0 | 0,18 | 0,005 |  | 0,18 | 0,003 |  | 0,18 | 0,007 |  | 0,18 | 0,022 |  | 0,20 | 0,019 |  |  |  |  |
| 5 | 0,18 | 0,005 |  | 0,18 | 0,004 |  | 0,19 | 0,016 |  | 0,18 | 0,016 |  | 0,20 | 0,027 |  |  |  |  |
| 10 | 0,17 | 0,008 |  | **0,17** | **0,002** | * | 0,17 | 0,018 |  | 0,17 | 0,019 |  | 0,20 | 0,020 |  |  |  |  |
| 15 | 0,16 | 0,005 |  | **0,16** | **0,002** | * | 0,18 | 0,024 |  | 0,16 | 0,015 |  | 0,18 | 0,013 |  |  |  |  |
| 20 | 0,16 | 0,003 |  | **0,16** | **0,008** | * | 0,17 | 0,017 |  | 0,15 | 0,008 |  | **0,17** | **0,019** | * |  |  |  |
| 25 | **0,15** | **0,004** | * | **0,14** | **0,006** | * | 0,16 | 0,016 |  | **0,13** | **0,009** | * | **0,14** | **0,012** | * |  |  |  |
| 30 | **0,14** | **0,010** | * | **0,12** | **0,005** | * | **0,12** | **0,006** | * | **0,11** | **0,009** | * | **0,12** | **0,013** | * |  |  |  |
| 40 | **0,10** | **0,003** | * | **0,07** | **0,005** | * | **0,07** | **0,015** | * | **0,06** | **0,011** | * | **0,06** | **0,014** | * |  |  |  |
| 50 | **0,07** | **0,015** | * | **0,04** | **0,002** | * | **0,03** | **0,021** | * | **0,03** | **0,006** | * | **0,03** | **0,006** | * |  |  |  |
| 75 | **0,00** | **0,001** | * | **0,00** | **0,001** | * | **0,00** | **0,000** | * | **0,00** | **0,000** | * | **0,00** | **0,000** | * |  |  |  |
| 125 | **0,00** | **0,003** | * | **0,00** | **0,003** | * | **0,00** | **0,000** | * | **0,00** | **0,000** | * | **0,00** | **0,000** | * |  |  |  |
